# Cytotoxicity and Antiviral Properties of Alkaloids Isolated from *Pancratium maritimum*

**DOI:** 10.3390/toxins14040262

**Published:** 2022-04-07

**Authors:** Marco Masi, Roberta Di Lecce, Natacha Mérindol, Marie-Pierre Girard, Lionel Berthoux, Isabel Desgagné-Penix, Viola Calabrò, Antonio Evidente

**Affiliations:** 1 Dipartimento di Scienze Chimiche, Università di Napoli Federico II, Complesso Universitario Monte Sant’Angelo, 80126 Napoli, Italy; marco.masi@unina.it (M.M.); roberta.dilecce@unina.it (R.D.L.); 2 Département de Chimie, Biochimie et Physique, Université du Québec à Trois-Rivières, Trois-Rivières, QC G8Z 4M3, Canada; natacha.merindol@uqtr.ca (N.M.); marie-pierre.girard@uqtr.ca (M.-P.G.); isabel.desgagne-penix@uqtr.ca (I.D.-P.); 3 Département de Biologie Médicale, Université du Québec à Trois-Rivières, Trois-Rivières, QC G8Z 4M3, Canada; lionel.berthoux@uqtr.ca; 4 Groupe de Recherche en Biologie Végétale, Université du Québec à Trois-Rivières, Trois-Rivières, QC G8Z 4M3, Canada; 5 Dipartimento di Biologia, Università di Napoli Federico II, Complesso Universitario Monte Sant’Angelo, 80126 Napoli, Italy; vcalabro@unina.it

**Keywords:** Amaryllidaceae, *Pancratium maritimum*, alkaloids, Dengue virus, human immunodeficiency virus, biological activity

## Abstract

Ten Amaryllidaceae alkaloids (AAs) were isolated for the first time from *Pancratium maritimum* collected in Calabria region, Italy. They belong to different subgroups of this family and were identified as lycorine, which is the main alkaloid, 9-*O*-demethyllycorine, haemanthidine, haemanthamine, 11-hydroxyvittatine, homolycorine, pancracine, obliquine, tazettine and vittatine. Haemanthidine was isolated as a scalar mixture of two 6-epimers, as already known also for other 6-hydroxycrinine alkaloids, but for the first time they were separated as 6,11-*O*,*O*′-di-*p*-bromobenzoyl esters. The evaluation of the cytotoxic and antiviral potentials of all isolated compounds was undertaken. Lycorine and haemanthidine showed cytotoxic activity on Hacat cells and A431 and AGS cancer cells while, pancracine exhibited selective cytotoxicity against A431 cells. We uncovered that in addition to lycorine and haemanthidine, haemanthamine and pancracine also possess antiretroviral abilities, inhibiting pseudotyped human immunodeficiency virus (HIV)−1 with EC50 of 25.3 µM and 18.5 µM respectively. Strikingly, all the AAs isolated from *P. maritimum* were able to impede dengue virus (DENV) replication (EC_50_ ranged from 0.34–73.59 µM) at low to non-cytotoxic concentrations (CC_50_ ranged from 6.25 µM to >100 µM). Haemanthamine (EC50 = 337 nM), pancracine (EC_50_ = 357 nM) and haemanthidine (EC_50_ = 476 nM) were the most potent anti-DENV inhibitors. Thus, this study uncovered new antiviral properties of *P. maritimum* isolated alkaloids, a significant finding that could lead to the development of new therapeutic strategies to fight viral infectious diseases.

## 1. Introduction

Plants belonging to the Amaryllidaceae family are distributed in Northern and Southern hemispheres, but they essentially grow in Andean South America, in the Mediterranean Basin, and in South Africa [1]. They are bulbous flowering plants including approximately 1600 species classified into about 75 genera [2]. These plants were used from ancient times for their beautiful flowers and essential oils. They are widely utilized in folk medicine because of the diverse and powerful therapeutic activities exhibited by their extracts and decoctions [3,4]. Many of these biological activities are due to their content in alkaloids that are present mostly in the plant bulbs [4,5,6].

The Amaryllidaceae alkaloids (AAs) are grouped in 12 ring types [7,8]. The extensive use of AAs producing plants in folk medicine and their varied carbon skeleton prompted a deep investigation into their potential as phytochemical-based drug discovery [9]. Hundreds of AAs with different carbon skeletons and functionalities have been isolated [1,5,6,8,9,10]. Among them, many have shown promising anticancer activity [11], as lycorine- and its related isocarbostyril narciclasine [12,13,14,15,16,17,18], crinine- [19,20,21], pretazettine- [22], and montanine- [23] type alkaloids. Many AAs also possess anti-acetylcholinesterase potential such as sanguinine isolated from *Crinum giganteum* [24] or galanthamine currently used as a cholinesterase inhibitor drug to treat Alzheimer’s disease [25]. Other AAs possess anti-parasital [26], anti-larvicidal and anti-insecticidal [27,28] properties. Some AAs display broad antiviral activity, i.e., lycorine, haemanthamine and 11-hydroxyvittatine inhibit the replication of influenza virus H5N1 [29], haemanthamine, lycorine and homolycorine were also shown to display antiretroviral activities [30], while lycorine and derivatives are potent inhibitors of flaviviruses and coronaviruses. Recently, we also showed that cherylline was a potent anti-flaviviral compound [31]. To date, there are no antiviral drugs approved to treat flaviviral infections such as dengue fever caused by the Dengue virus (DENV) that affects 400 million people each year. Moreover, despite a highly efficacious combined antiretroviral therapy, there are still no curative agents for the 37.87 million people living with HIV-1 that must take their medication all their life to prevent viral replication. Thus, the characterization of AAs antiviral activities is at the front line for new drug discovery.

Recent studies emphasized the importance of investigating either uncharacterized species or known species but collected in different regions of the world to grasp Amaryllidaceae’s diverse potential as a source of AAs.

Studies on the alkaloids produced by *Pancratium maritimum* started in 1954 with the investigation performed by du Merac (1954) [32]. Subsequently, investigations allowed to isolate alkaloids belonging to the subgroups lycorine-, lycorenine-, montanine- tazettine- galanthamine- haemanthidine- and crinine-, isocarbostiryl as narciclasine- and pancratistatin-type [33]. In particular, 40 alkaloids were isolated from bulbs and leaves extracts of *P. maritimum* collected in Turkey, Egypt, Italy (Apulia region) and Bulgaria, showing purgative, acaricidal, insecticidal, and antifungal activities [34]. Among them, (-)-3b,11a-dihydroxy-1,2-dehydrocrinane and (-)-8-hydroxy-9-methoxycrinine trisphaeridine, pancrimatines A, B and C, and norismine, were isolated [35]. In other studies, ungeremine and zefbetaine were isolated [36], as well as narciclasine-4-*O*-β-D-glucopyranoside [37]. Ungeremine exhibited significant bactericide activity against the Gram-negative *Flavobacterium columnare* causing channel catfish (*Ictalurus punctatus*) columnaris disease [38]. Furthermore, ungeremine was bioformulated in chitosan tripolyphosphate which was particularly active against *Penicillium roqueforti*, a fungus responsible for bakery products deterioration [39,40].

**Figure 1:**
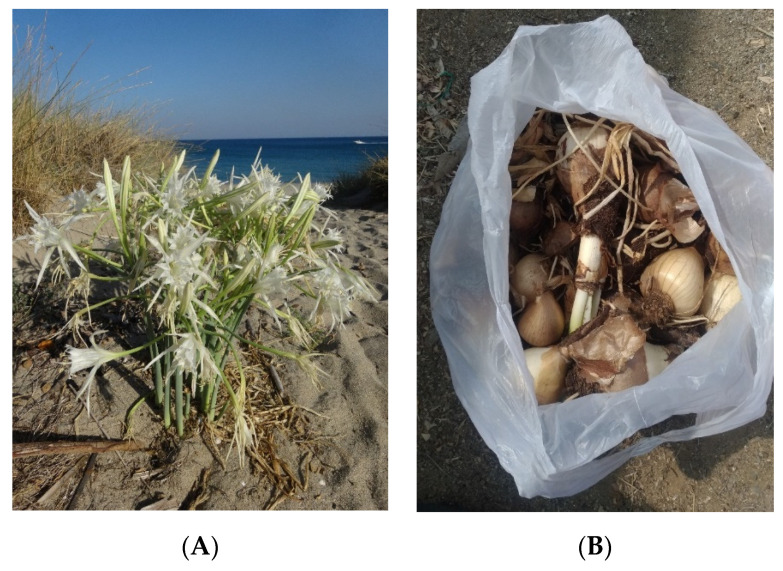
Flowers (**A**) and bulbs (**B**) of *Pancratium maritimum*.

Considering that organic plant extracts show a high level of variability in their composition depending on several factors (e.g., geographical origin, inter- and intra-specific genetic variability, plant phenologic stage), and the need for new therapeutics to treat human diseases, a new investigation of the alkaloid content of *P. maritimum* collected for the first time in Calabria region, Italy, was carried out (Figure 1).

This study reports the identification of ten alkaloids isolated from the acid bulb extract of *P. maritimum* and for the first time describes the separation of the two 6-epimers of haemanthidine as *p*-bromobenzoyl derivatives, which also allowed us to assign their relative and absolute configuration. The results of their cytotoxic and antiviral properties were also described.

## 2. Results and Discussion

**Figure 2:**
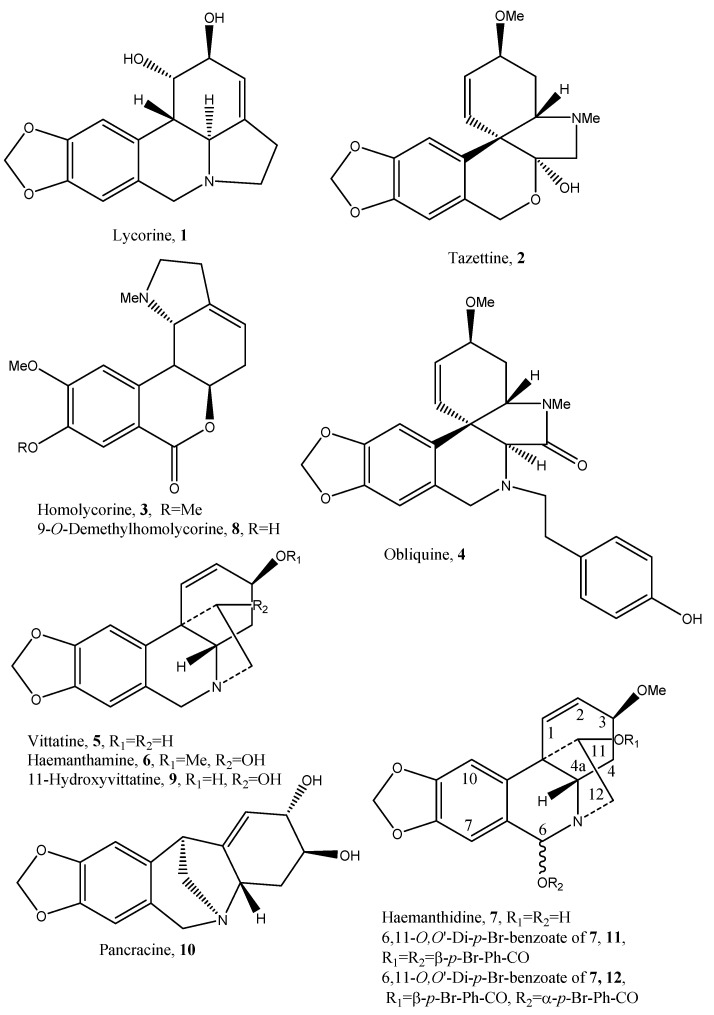
Structures of lycorine, tazettine, homolycorine, obliquine, vittatine, haemanthamine, haemanthidine, 9-*O*-demethylhomolycorine, 11-hydroxyvittatine, pancracine (**1**–**10**) isolated from *P. maritimum* and that of the two diastereomeric *p*-bromobenzoyl esters of **7** (**11** and **12**).

Dried and minced *P. maritimum* bulbs were acid extracted and the aqueous phase was re-extracted with EtOAc following alkalinization, as detailed in Experimental section. Evaporation of the solvent left an oily residue which was crystallized and yielded an abundant amount of lycorine (**1**, Figure 2), the main isolated metabolite. The mother liquors resulting from lycorine crystallization were evaporated under reduced pressure, yielding a residue which was purified using a combination of column and TLC chromatography on silica gel, allowing us to isolate ten already known AAs identified as tazettine, homolycorine, obliquine, vittatine, haemanthamine, haemanthidine, 9-*O*-demethylhomolycorine, 11-hydoxyvittatine and pancracine (**2**–**10**, Figure 2), thus belonging to lycorine- (**1**), pretazettine- (**2**), homolycorine- (**3**, **4** and **8**), crinine- (**5**–**7** and **9**) and pancracine- (**10**) types.

These alkaloids were identified comparing their physical (specific optical rotation) and spectroscopic (^1^H and ^13^C NMR and ESI MS) properties with those already reported in the literature: for **1** by Lamoral-Theys et al. (2009) [13] isolated from *Stenbergia lutea*, for **2** with those reported by Antoun et al. (1993) [41] and Boit and Döpke (1960) [42] when isolated from *Hymenocallis expansa* and *Hippeastrum aulicum*, respectively. Alkaloids **3** and **4** were identified by comparing our experimental results with those reported by Fales et al. (1956) [43], Furosawa et al. (1976) [44] and Baxendale and Lei (2005) [45] when isolated from *Narcissus tazetta* and *Cyrtanthus obliquus*. Alkaloids **5** and **6** were identified comparing our data with those reported by Frahm et al. (1985) [46], Bastida et al. (1995) [47], Pabuççuoglu et al. (1989) [48] and Ghosal et al. (1985) [49] when isolated from *Crinum asiaticum*, *Narcissus cantabricus* and *Sternbergia sicula*, respectively. Likewise, alkaloids **7** and **8** were identified by comparing their data with those described by Ma et al. (1986) [50], Antoun et al. (1993) [41], Forgo and Hoffmann (2005) [51], Bastida et al. (1987) [52] and Evidente et al. (1994) [53] when isolated from *Hymenocallis expansa, Narcissus tazetta* L. var *chinensis roem, Leucojum vernum* and *Narcissus confusus*, respectively. Finally, alkaloids **9** and **10** were identified by comparison with those reported by Evidente (1986) [54] and Ali et al. (1984) [55] when isolated from *Hippeastrum vittatum* and *Stenbergia lutea*, respectively. Interestingly, among all the alkaloids isolated in this study, obliquine (**4**) and pancracine (**10**) were isolated for the first time from *P. maritimum*.

As previously reported, haemanthidine (**7**) was isolated as two 6-epimers [56] in a scalar ratio. The equilibrium between the two epimers was also reported for 6-hydroxycrinamine, but not for closely related crinine-type alkaloids such as 6α-hydroxycrinine and 6α-hydroxybuphonisine. The equilibrium between the two epimers could be explained through their interconversion in solution via the corresponding aminoaldehyde [57]. The ratio of the two epimers in solution, evidenced by ^1^H NMR spectroscopy, is affected from the variable temperature at which the spectrum is recorded. Haemanthidine (**7**) epimeric ratio was of 2:1, whereas King et al. (1965) [57] observed a 1:1 ratio. Any attempt to separate the two 6-epimers and to determine their absolute configuration failed. In addition, their esters were not separable upon converting the mixture of 6-epimers in the corresponding 6,11-*O*,*O*′-diacetyl derivatives. Instead, when converted by reaction with *p*-bromobenzoyl chloride in the usual conditions, haemanthidine yielded two corresponding epimeric esters (**11** and **12**, Figure 2) which were easily separated by TLC chromatography and characterized by physic properties (specific optical rotation), ^1^H NMR and ESI MS spectroscopy. The comparison of the two ^1^H NMR spectra (Table 1) of **11** and **12** with that of **7** showed as substantial difference the presence of the signal pattern of a *p*-disubstituted phenyl residue, appearing as two couples of doublets (*J* = 8.2 and *J* = 8.6 Hz) at δ 7.93 and 7.60 (2′,6′ and 3′,5′) and 7.77 and 7.58 (2″,6″ and 3″,5″) for **11**, and (*J* = 8.4 and *J* = 8.7 Hz) at δ 8.00 and 7.62 (2′,6′ and 3′,5′), and 7.77 and 7.59 (2″,6″ and 3″,5″) for **12**.

Furthermore, the expected significant downfield shifts H-6 and H-11 were observed. In particular, in the spectrum of **11**, H-6 and H-11 appeared as a singlet and a double doublets (*J* = 7.6 and 3.6 Hz) downfield shifted (Δδ 1.27 and 1.23) with respect to **7** at δ 6.38 and 5.18, similarly in **12**, the same signal appeared as a singlet (H-6) and a double doublet (*J* = 6.9 and 2.7 Hz) (H-11) downfield shifted (Δδ 1.18 and 1.25) with respect to **7** at δ 6.93 and 5.20, respectively.

Furthermore, **11** and **12**, as expected showed the same typical ESI MS spectrum exhibiting the three peaks due the presence of the two isotopes of the two bromine at *m*/*z* 696 [M + H]^+^, 698 [M + 2 + H]^+^, 700 [M + 4 + H]^+^.

The NOESY spectra (Table 2) of **11** and **12** showed both the expected correlations of H-1 with H-10 and H-2, H-2 with H-3, the latter with H_2_-4 and MeO, H-11 with H-12A and the latter with H-12B. There was a significant difference with regards to the correlation observed in **12** between H-6 and H-4a, which was absent in **11**. This last result allowed us to assign the same β-orientation of H-6 as H-4a in **12**, while in **11** H-6 resulted to be α-located. Thus, knowing the absolute configuration of other chiral centers of haemanthidine [58], H-6 has a *S*-configuration in **12** and an *R*-configuration in **11**. All the attempts to re-covert the *p*-bromobenzoyl esters in the free base **7** by hydrolysis carried out in different conditions failed.

Previous literature reported that several AAs, including haemanthidine as a 6-epimer mixture displayed cytotoxic potential against several types of cancer cell lines [59] and the growth inhibitory effect on both parental and multidrug resistant L5178 mouse lymphoma cell lines [60]. Thus, the cytotoxic activity was assessed for all the isolated alkaloids (**1**–**6** and **8**–**10**), the scalar 6-epimer mixture of **7** and their two ester **11** and **12**.

**Figure 3:**
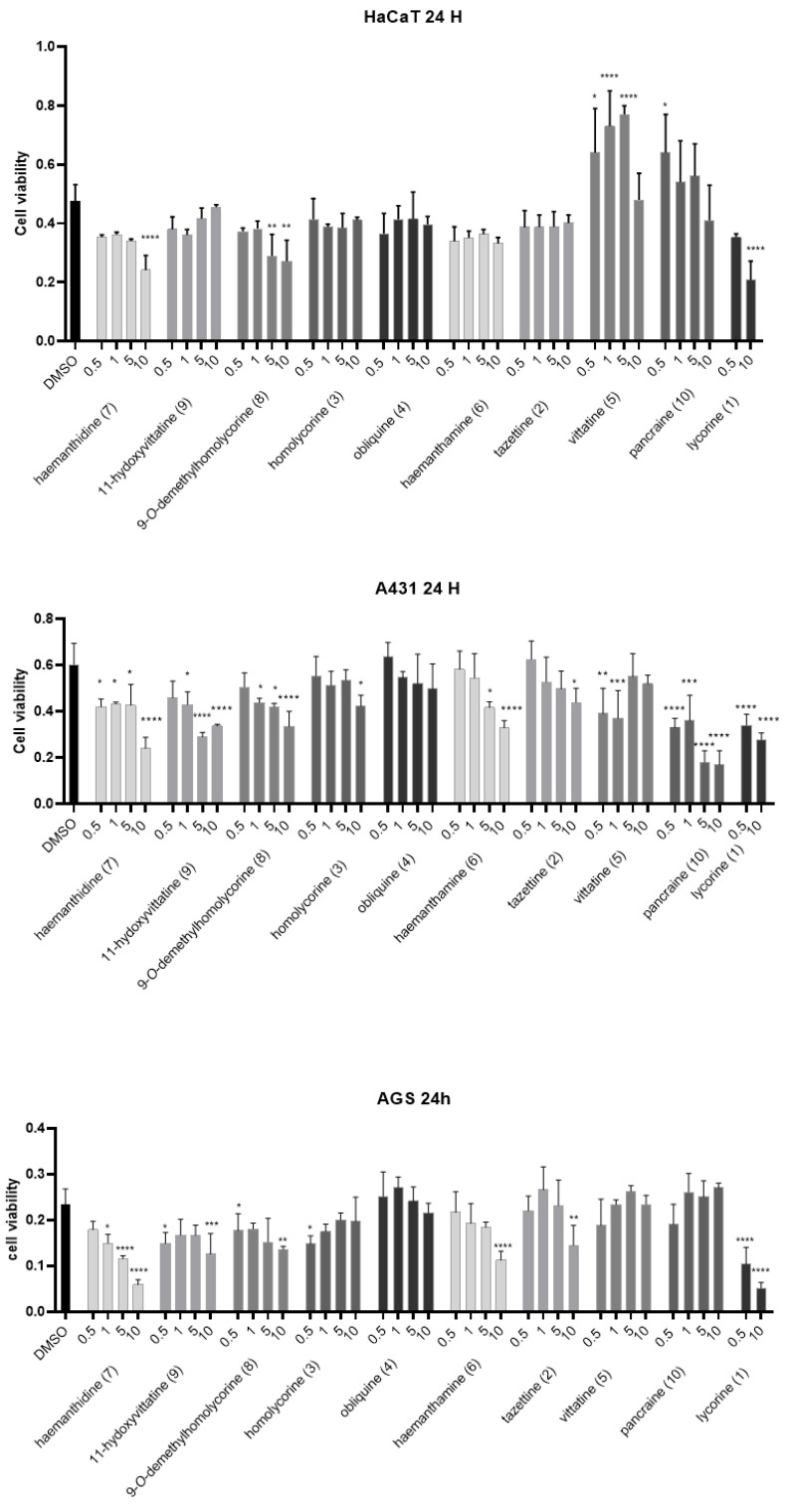
MTT viability test. Hacat, A431 and AGS cells were incubated with the indicated amounts of alkaloids in DMSO, for 24 h. The values were the mean’s six values for each experimental point of biological replicates. Each mean was compared using a Dunnett’s multiple comparisons test of ANOVA one-way (*p*-value * < 0.01, ** < 0.05, *** *p* < 0.001; **** *p* < 0.0001). DMSO control.

**Figure 4:**
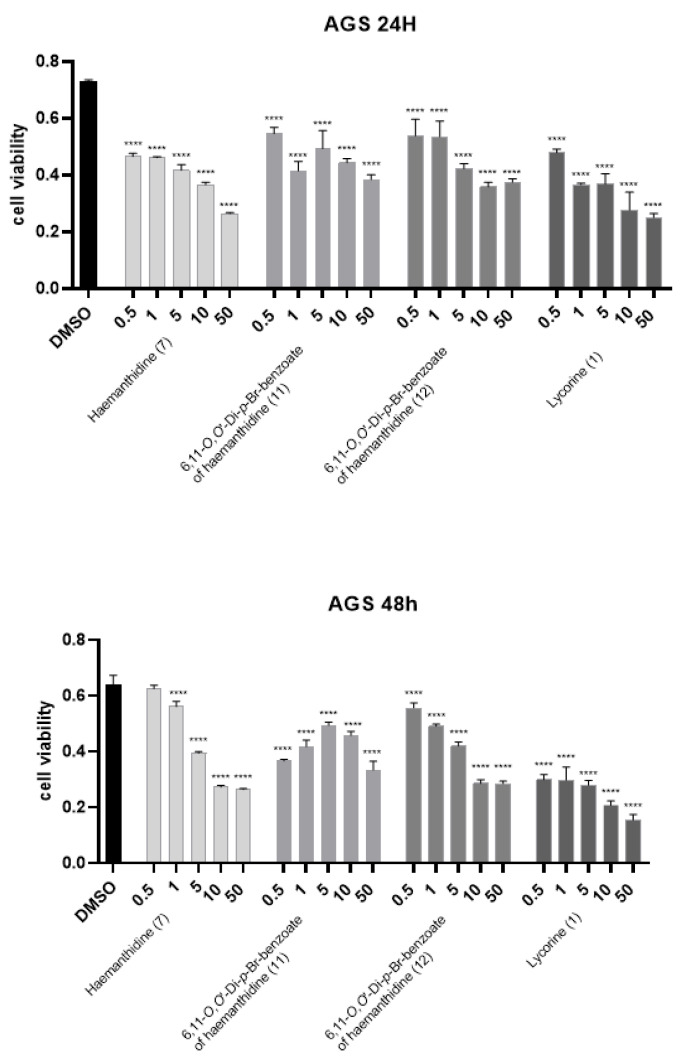
MTT viability test of *P. maritimum* alkaloid. AGS cells were incubated with the indicated amount of haemantidine (**7**), β-epimer (**11**). α-epimer (**12**), and lycorine (**1**) in DMSO, for 24 h (upper panel) or 48 h (lower panel). The values were the mean’s six values for each experimental point of biological replicates. Each mean was compared using a Dunnett’s multiple comparisons test of ANOVA one-way (*p*-value **** *p* < 0.0001). DMSO control.

The potential cytotoxic activity of *P. maritimum* alkaloids was evaluated on Hacat, A431 and AGS cells treated with concentrations ranging from 0.5 to 10 µM for 24 and 48 h by measuring the reduction of 3-(4,5-dimethylthiazol-2) 2,5- diphenyltetrazolium bromide (MTT) to formazan by the mitochondrial enzyme succinate dehydrogenase. We selected Hacat cells because they are spontaneously immortalized human keratinocytes and they have been used in several studies as a paradigm for non-transformed epidermal cells. A431 and AGS cells were chosen as transformed epithelial cells derived from skin epidermoid tumor and gastric carcinoma, respectively. Interestingly, as shown in Figure 3 and Figure 4, in addition to lycorine (IC_50_ < 0.5 µM), AGS cells were sensitive to haemanthamine (**6**) (IC_50_ = 7.5 µM at 24 and 48 h) and haemanthidine (**7**) (IC_50_ = 5.0 µM at 24 h), albeit at much higher concentrations (Table 3).

We further evaluated the cytotoxic activity of the two *p*-bromobenzoyl derivatives (**11**, **12**) of haemanthidine with concentrations ranging from 0.5 to 50 µM for 24 and 48 h. As shown in Figure 4, both epimers were able to reduce AGS cell viability.

The only alkaloid with cytotoxic activity on Hacat and A431 cells was lycorine (IC_50_ = 0.5 µM at 48 h for both cell lines). Indeed, we only observed a slight to moderate reduction of Hacat and A431 cell viability (between 15–30%) with 10 µM haemanthidine, -*O*-demethylhomolycorine, haemanthamine and tazettine at 24 h of treatment. At 48 h, cell growth was almost completely rescued suggesting that cells underwent a reversible cell cycle arrest. This suggest that haemanthidine and haemanthamine could display a selective toxicity towards cancer cells.

**Figure 5:**
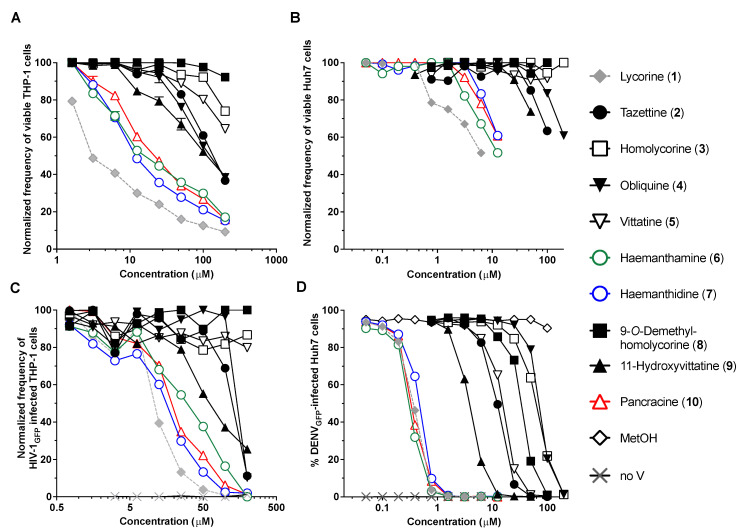
Antiviral assays. (**A**) Cytotoxicity of *P. maritimum’s* isolated compounds measured in THP-1 cells. (**B**) Cytotoxicity in Huh7 cells. Cell viability was assessed by measuring ATP levels. The frequency of viable cells is expressed as a percentage of ATP levels in cells treated with alkaloids normalized to cells treated with matched concentrations of vehicle methanol. Wells without cells were used as negative controls. (**C**) Anti-retroviral potential of isolated compounds. Alkaloid-treated THP-1 cells were infected with HIV-1_GFP_ at an MOI = 0.1. Infection levels were measured 72 h later and normalized using methanol treated cells. Raltegravir (10 µM) used as a positive control completely impeded HIV-1 infection. (**D**) Anti-flaviviral potential of isolated compounds. DENV_GFP_ (MOI = 0.02) was used to infect Huh7 cells treated with alkaloids. Infection levels were measured 72 h later. Shown are representative results of three independent experiments.

Among the alkaloids isolated from *P. maritimum* in this study, several have been associated with antiviral functions, while others have not been tested. Lycorine, haemanthamine and 11-hydroxyvittatine were reported to inhibit the replication of influenza virus H5N1 [29]. Haemanthamine, lycorine and homolycorine were also shown to display antiretroviral activities [30], while lycorine and derivatives are potent inhibitors of flaviviruses and coronaviruses. In this study, we assessed the antiviral activity of all the isolated alkaloids (**1**–**6** and **8**–**10**) and the scalar 6-epimer mixture of **7**. We focused on the antiretroviral and anti-flaviviral activities, as we and others have previously shown that several AAs efficiently target HIV-1 and DENV replication (Figure 5).

To account for non-specific antiviral activity caused by enhanced cell death, the cytotoxicity of *P. maritimum* isolated AAs was first determined in THP-1 and Huh7 virus-targeted cell lines exposed to concentrations ranging from 0.05 to 200 µM for 72 h. Cell viability was measured using ATP levels as a marker. The most cytotoxic compounds were lycorine, haemanthamine, pancracine, haemanthidine, followed by 11-hydroxyvitattine, tazettine and obliquine in both THP-1 and Huh7 cells (Figure 5A,B). In THP-1 cells used for HIV-1 infection, lycorine (CC_50_ = 4.6 µM) impacted cell viability at all concentrations whereas haemanthidine (CC_50_ = 16.8 µM), haemanthamine (CC_50_ = 22.2 µM) and pancracine (CC_50_ = 25.93 µM), decreased cell viability beginning at 3.125 µM with 71%, 71% and 82% of live cells at 6.25 µM, respectively. A concentration of 100 µM of obliquine, tazettine and 11-hydroxyvittatine reduced viable THP-1 cells to 55.3%, 60.9% and 52.3%, respectively (Figure 5A and Table 3). In the Huh7 cells used for DENV infection, 0.78 µM of lycorine reduced viability by 21%, while haemanthamine was cytotoxic at concentrations above 3.125 µM (82.2% live cells), and pancracine and haemanthidine decreased viability beginning at 6.25 µM (78.1% and 83.3% of live cells, respectively). 11-hydoxyvittatine and tazettine reduced viability at concentrations higher than 50 µM (73.8% and 85.2% viable cells, respectively) and obliquine was toxic from 100 µM (82.6%) (Figure 5B). The AAs CC_50_ were not reached at the concentrations used in our assays.

Next, pseudotyped HIV-1_GFP_ vectors were used to assess the antiretroviral potential of alkaloids. These vectors enter THP-1 cells, reverse transcribe their viral RNA, integrate viral DNA into the host genome, produce viral proteins and GFP, but do not lead to the production of infectious viral particles. We treated THP-1 cells with each AA and infected them at an MOI = 0.1 (Figure 5C). The percentage of infected GFP^+^ THP-1 cells was measured 72 h post-infection using a flow cytometer. Lycorine, haemanthamine, pancracine, haemanthidine, 11-hydroxyvittatine, tazettine and obliquine displayed a dose-dependent inhibition of HIV-1. Complete viral inhibition was observed for haemanthidine, lycorine, haemanthamine, and pancracine at 100 µM (Figure 5C) with EC_50_ of 12.7 µM, 10.9 µM, 25.3 µM, 18.5 µM, respectively (Figure 5C and Table 4). Whereas haemanthamine and lycorine have been previously reported to display antiretroviral activities [30], our results suggest that haemanthidine and pancracine also share this function. However, and for all four alkaloids, only 40–50% of cells remain viable at the EC_50_. Thus, although these alkaloids display interesting antiretroviral effects as previously reported, the effective concentrations to block infection are considerably cytotoxic.

Lycorine is recognized for its anti-flaviviral properties. We assessed AAs’ anti-flavivirus properties using the DENV_GFP_ dengue vector, which is propagation-competent. Upon infection, this vector leads to the production of GFP concomitantly with the translation of viral genomic RNA. Infection was measured by assessing the % of GFP^+^ cells 72 h post-infection on a flow cytometer (Figure 5D). Obliquine (**4**) (EC_50_ = 73.6 µM), homolycorine (**3**) (EC_50_ = 65.0 µM), 9-*O*-demethylhomolycorine (**8**) (EC_50_ = 34.49 µM), vittatine (**5**) (EC_50_ = 15.53 µM), tazettine (**2**) (EC_50_ = 12.5 µM), 11-hydroxyvittatine (**9**) (EC_50_ = 3.92 µM), haemanthidine (**7**) (EC_50_ = 0.476 µM), lycorine (**1**) (EC_50_ = 0.386 µM), pancracine (**10**) (EC_50_ = 0.358 µM), and haemanthamine (**6**) (EC_50_ = 0.337 µM) all impeded DENV_GFP_ replication. Complete inhibition was obtained at 1.56 µM for lycorine, haemanthamine, pancracine and haemanthidine. At this concentration, only lycorine was cytotoxic at detectable levels, reducing cell viability by 25% (Figure 5B,D). To our knowledge, this is the first report on these AAs inhibiting flavivirus at levels comparable to lycorine and less cytotoxicity. Thus, we have uncovered that *Pancratium maritimum*’s extracted haemanthamine, haemanthidine and pancracine are potent antiflaviviral compounds that should be further explored.

## 3. Conclusions

This manuscript reports an in-depth investigation of *P. maritimum* isolated from the first time in South Italy Coast. Ten already known Amaryllidaceae alkaloids belonging to different subgroups were identified as lycorine, the main alkaloid, 9-*O*-demethyllycorine, haemanthidine, haemanthamine, 11-hydroxyvittatine, homolycorine, pancracine, obliquine, tazettine and vittatine, with obliquine and pancracine isolated for the first time from this Amaryllidaceae. Lycorine and haemanthidine showed cytotoxic activity on Hacat cells and A431 and AGS cancer cells while, pancracine exhibited selective cytotoxicity against A431 cells. Finally, we uncovered that *P. maritimum*’s isolated haemanthamine, haemanthidine and pancracine display promising antiflaviviral activity.

## 4. Materials and Methods

### 4.1. General Experimental Procedures

Optical rotations were measured in a MeOH solution on a Jasco P-1010 digital polarimeter (Jasco, Tokyo, Japan). ^1^H and ^13^C NMR spectra were recorded at 400 and 100 MHz, respectively, in CD_3_OD or otherwise noted on a Bruker spectrometer (Bruker, Karlsruhe, Germany). The same solvent was used as an internal standard. and Nuclear Overhauser Effect Spectroscopy (NOESY) experiments [61] were performed using Bruker microprograms; ESI mass spectra and liquid chromatography (LC)/MS analyses were performed using the LC/MS TOF system Agilent 6230B, HPLC 1260 Infinity (Agilent, Milam, Italy). The HPLC separations were performed with a Phenomenex LUNA (C18 (2) 5 μ 150 × 4.6 mm. Analytical and preparative Thin-Layer Chromatography (TLC) was performed on silica gel plates (Merck, Darmstadt, Germany, Kieselgel 60, F254, 0.25 and 0.5 mm, respectively) or on reverse phase (Whatman, Maidstone, UK, KC18 F254, 0.20 mm) plates and the compounds were visualized by exposure to UV light and/or iodine vapors CC: silica gel (Merck, Darmstadt, Germany, Kieselgel 60, 0.063–0.200 mm).

### 4.2. Plant Material

Bulbs of *Pancratium maritimum* were collected in Squillace, Italy (38°47′20.6″ N and 16°35′10.8″ W), in August 2020 (Figure 1). The plant specimen is deposited in the collection of the same Department of Biology, University of Naples Federico II.

### 4.3. Extraction and Purification of Alkaloids

Fresh bulbs of *P. maritimum* (6 kg) were dried at room temperature and then finely powdered. The resulting powder (736.2 g) was extracted with 1% H_2_SO_4_, (2 × 1 L) overnight at room temperature. The suspension was filtered through cloth and successively centrifuged at 10 °C at 7000 rpm for 30 min. The acid extract was alkalinized to pH 9–10 with 12 N NaOH. The aqueous solution was extracted with EtOAc (3 × 1 L), and the organic extracts were combined, dried (Na_2_SO_4_) and evaporated under reduced pressure to give a brown oil residue (3.9 g). This latter was crystallized by EtOH obtaining lycorine (**1**, 900 mg) as white crystals. The mother liquors of crystallization were dried, and the residue (3.0 g) was fractionated by column chromatography eluted with CHCl_3_-EtOAc-MeOH (2:2:1), affording eight groups of homogeneous fractions (F1–F8). The residue (182.5 mg) of fraction F3 was purified on silica gel column chromatography (CC) eluted with CHCl_3_-*i*-PrOH (9:1), yielding five fractions (F3.1–F3.5). The residue (50.5 mg) of fraction 2 resulted to be an amorphous solid identified as tazettine (**9**). The residue (336.5 a mg) of fraction F4 was purified on CC eluted with EtOAc-MeOH-H_2_O (7:2:1) yielding seven homogeneous fractions (F4.1–F4.7). The residue (48.9 mg) of F4.1 was further purified by preparative TLC eluted with CHCl_3_-EtOAc-MeOH (2:2:1) obtaining an amorphous solid identified as homolycorine (**6**, 8.4 mg). The residue (15.6 mg) of F4.3 was purified by TLC eluted with CHCl_3_-EtOAc-MeOH (2:2:1) yielding obliquine (**8**, 4.6 mg) as an amorphous solid. The residue (546.6 mg) of F5 was purified by CC eluted with EtOAc-MeOH-H_2_O (7:2:1) yielding six homogeneous fractions (F5.1–F5.6). The residue (67.5 mg) of fraction F5.2 was purified on preparative TLC with CH_2_Cl_2_-MeOH (8:2) yielding vittatine (**4**, 8.6 mg) and haemanthamine (**3**, 2.2 mg) as amorphous solids. The residue (97.5 mg) of fraction F5.3 was purified on preparative TLC with CHCl_3_-EtOAc-MeOH (6:2:2) yielding haemanthidine (**2**, 30.6 mg) as an amorphous solid. The residue (12.9 mg) of fraction F5.5 was purified on preparative TLC with CHCl_3_-*i*-PrOH (7:3) yielding an amorphous solid identified as 9-*O*-demethylhomolycorine (**7**, 3.3 mg). The residue (333.5 mg) of F6 was purified by CC eluted EtOAc-MeOH-H_2_O (7:2:1), yielding five groups of homogeneous fractions (F6.1–F6.5). The residue (62.6 mg) of fraction F6.3 was purified by preparative TLC eluted twice with CHCl_3_-EtOAc-MeOH (2:2:1) obtaining an amorphous solid identified as 11-hydroxyvittatine (**5**, 13.4 mg). The residue (31.2 mg) of fraction F6.4 was purified by TLC eluted with EtOAc-MeOH-H_2_O (7:2:1) obtaining an amorphous solid identified as pancracine (**10**, 9.4 mg).

Lycorine (**1**). [α]_D_^25^ −72.5 (c 0.1, CH_3_OH) [lit. [62]: [α]_D_^25^ −71.2 (c 0.1, CH_3_OH)]; ^1^H and ^13^C NMR data are in agreement to those previously reported [13]. ESIMS (+) *m*/*z*: 288 [M + H]^+^.

Tazettine (**2**). [α]_D_^25^ +143.1 (c 1.2, CHCl_3_) [lit. [41]: [α]_D_^25^ +138 (c 0.23, CHCl_3_)]; ^1^H NMR (CD_3_OD) data are in agreement to those previously reported [42]. ESIMS (+) *m*/*z*: 332 [M + H]^+^.

Homolycorine (**3**). [α]_D_^25^ +95.3 (c 1.2, CHCl_3_) [lit. [43]: [α]_D_^26^ +93.5 (c 1.2, CHCl_3_)]; ^1^H NMR (CD_3_OD) data are in agreement to those previously reported [44]. ESIMS (+) *m*/*z*: 316 [M + H]^+^.

Obliquine (**4**). [α]_D_^25^ −135.6 (c 0.1, CH_3_OH) [lit. [45]: [α]_D_^25^ −137.8 (c 0.3, CH_3_OH)]; ^1^H and ^13^C NMR (CDCl_3_) data are in agreement to those previously reported [45]. ESIMS (+) *m*/*z*: 449 [M + H]^+^.

Vittatine (**5**). [α]_D_^25^ +28.0 (c 1.2, CHCl_3_) [lit. [47]: [α]_D_^20^ +26 (c 0.25, CH_3_OH)]; ^1^H NMR (CD_3_OD) data are in agreement to those previously reported [46,48]. ESIMS (+) *m*/*z*: 272 [M + H]^+^.

Haemanthamine (**6**). [α]_D_^25^ +41.0 (c 1.2, CHCl_3_) [lit. [49]: [α]_D_^22^ +38.3 (c 0.45, CHCl_3_)]; ^1^H NMR (CD_3_OD) data are in agreement to those previously reported [46,48]. ESIMS (+) *m*/*z*: 302 [M + H]^+^.

Haemanthidine (**7**). [α]_D_^25^ −12.2 (c 1.2, CHCl_3_) [lit. [41]: [α]_D_^25^ −14 (c 0.28, CHCl_3_)]; ^1^H and ^13^C NMR (CDCl_3_) data are in agreement to those previously reported [50]. ESIMS (+) *m*/*z*: 318 [M+H]^+^.

9-*O*-Demethylhomolycorine (**8**). [α]_D_^25^ +56.1 (c 0.1, CHCl_3_) [lit. [51]: [α]_D_^25^ +57 (c 0.2, CHCl_3_)]; ^1^H NMR (CD_3_OD) data are in agreement to those previously reported [52,53]. ESIMS (+) *m*/*z*: 302 [M + H]^+^.

11-Hydroxyvittatine (**9**). [α]_D_^25^ +12.1 (c 0.1, CH_3_OH) [lit. [54]: [α]_D_^25^ +11.3 (c 0.88, CH_3_OH)]; ^1^H NMR (CD_3_OD) data are in agreement to those previously reported [54]. ESIMS (+) *m*/*z*: 288 [M + H]^+^.

Pancracine (**10**). [α]_D_^25^ +72.9 (c 0.1, CH_3_OH) [lit. [55]: [α]_D_^25^ +74 (c 0.6, CH_3_OH)]; ^1^H and ^13^C NMR (CD_3_OD) data are in agreement to those previously reported [55]; ESIMS (+) *m*/*z*: 288 [M + H]^+^.

### 4.4. Conversion of Haemanthidine in the Corresponding 6,11-O,O′-di-*p*-Bromobenzoyl Esters (***11*** and ***12***) 

Hemanthidine, (**7**) (2.0 mg), was dissolved in CH_3_CN (600 µL), and 4-dimethylaminopyridine (DMAP) (4.3 mg) and p-bromobenzoyl chloride (4.1 mg) were added. The reaction mixture was stirred at room temperature for 24 h and then evaporated under reduced pressure. The residue (10.2 mg) was purified by TLC on silica gel, eluent n-hexane-EtOAc (6:4) giving Derivatives **11** (2.3 mg), and **12** (3.8 mg), as colorless oils. **11** had: ^1^HNMR, See Table 1; ESI-MS (+) *m*/*z* 696 [M + H]^+^, 698 [M + 2 + H]^+^, 700 [M + 4 + H]^+^. **12** had: ^1^H NMR, See Table 1; ESI-MS (+) *m*/*z* 696 [M + H]^+^, 698 [M + 2 + H]^+^, 700 [M + 4 + H]^+^.

### 4.5. Biological Assays

#### 4.5.1. Antiviral Assays

Vesicular-stomatitis-virus G protein (VSV-G)-pseudotyped human immunodeficiency virus-1 [63] and replicative dengue virus (obtained from Ralf Bartenschlager) [64] vectors, both expressing the green fluorescent protein (GFP), were used to investigate alkaloids’ antiviral properties. The multiplicity of infection (MOI) of HIV-1_GFP_ was assessed by measuring the infectivity of serially diluted vector preparation in Crandell-Rees Feline Kidney (CRFK) cells, while DENV_GFP_ titer was measured by plaque assay in VERO cells.

The monocytic cell line THP-1 derived from an acute monocytic leukemia patient was used in HIV-1_GFP_ infection assays, while the hepatocyte-derived carcinoma cell line Huh7 was used with DENV_GFP_. Cell viability was evaluated using the Cell-Titer GLO assay kit (Promega, Madison, WI, USA) as previously described [31]. Briefly, 7.5 × 10^3^ Huh7 cells/well or 4 × 10^4^ THP-1 cells/well were seeded in 96-well dark plates and treated with isolated AAs dissolved in methanol at concentrations ranging from 0.05 to 200 µM for 72 h. Afterwards, Cell-Titer GLO reagent was added in each well of room temperature-equilibrated plates. Luminescence was measured 10 min after shaking the plates for 2 min using a microplate spectrophotometer (Synergy H1, Biotek, Québec, QC, Canada). The percentage of viable cells was calculated at each concentration relatively to cells treated with matched concentrations of MetOH.

To quantify HIV-1_GFP_ and DENV_GFP_ replication inhibition, 4.0 × 10^4^ THP-1 and 7.5 × 10^3^ Huh7 seeded cells per well were treated with indicated concentrations of alkaloids (from 0.05 to 200 µM) and then infected with HIV_GFP_ at MOI = 0.1 or DENV_GFP_ at MOI = 0.02. After 72 h, the percentage of infected cells was measured using a FC500 MPL cytometer (Beckman Coulter, Inc., Brea, CA, USA) and analyzed with the FlowJo software (BD, FlowJo LLC, Ashland, OR, USA). Matched concentrations of methanol were used as a negative control. Raltegravir was used as a positive control of HIV-1 inhibition, while lycorine was the positive control of DENV inhibition. All experiments were performed three times. CC_50_ and EC_50_ values were calculated using QuestGraph IC50 (MLA, Quest Graph™ IC50 Calculator”. AAT Bioquest, Inc., Sunnyvale, CA, USA, https://www.aatbio.com/tools/ic50-calculator (accessed on 18 December 2021)).

#### 4.5.2. Cell Culture and Reagents

HaCaT, spontaneously immortalized keratinocytes from adult skin, and AGS gastric adenocarcinoma cells were purchased from Service Cell Line (GmBH, Eppelheim, CLS, Germany). A431 (ATCC-CRL1555) human epidermoid carcinoma cells were from American Type Culture Collection (ATCC, Manassas, VA, USA). All mentioned cell lines were cultured in Dulbecco’s Modified Eagle’s Medium (DMEM, Sigma Chemical Co., St. Louis, MO, USA) supplemented with 10% fetal bovine serum (FBS, Hyclone Laboratories, Inc. Logan, UT, USA) at 37 °C in a humidified atmosphere of 5% CO_2_. Absence of mycoplasma contamination was verified for all cell lines. The purified alkaloids were resuspended in DMSO and added to the cell medium at concentrations varying from 0.5 to 50 µM as indicated.

#### 4.5.3. MTT Assay

Cell viability was evaluated by measuring the reduction of 3-(4,5-dimethylthiazol-2) 2,5-diphenyltetrazolium bromide (MTT) to formazan by the mitochondrial enzyme succinate dehydrogenase. Briefly, 10 × 10^3^ cells were seeded on 96-well plates and exposed to different concentrations of total extract or metabolites for 48 h. and 72 h. MTT/PBS solution (0.5 mg/mL) was then added to the wells and incubated for 3 h at 37 °C in a humidified atmosphere. The reaction was stopped by removal of the supernatant followed by dissolving the formazan product in acidic isopropanol and the optical density was measured with Synergy H4 microplate reader Gen5 2.07 (Thermofisher, Waltham, MA, USA) using a 570 nm filter. Under these experimental conditions, no undissolved formazan crystals were observed. Cell viability was assessed by comparing the optical density of the treated samples compared to the controls. Statistical analyses were carried out using the GraphPad Prism version 8.1.2 (https://www.graphpad.com/scientific-software/prism/ (accessed on 18 December 2021)). Data were represented as the mean standard deviation and analyzed for statistical significance using ordinary one-way analysis of variance (ANOVA) and multiple comparisons. For all tests, *p* < 0.05 was considered to indicate a statistically significant difference.

## Figures and Tables

**Table 1:** ^1^H NMR Data of the two 6,11-*O*,*O*′-Di-*p*-Bromobenzoyl Esters (**11** and **12**) ^a,b^ of haemanthidine (**7**).

	11	12
**No.**	δ_H_ (*J* in Hz)	δ_H_ (*J* in Hz)
1	6.40 d (10.4)	6.39 d (10.1)
2	6.15 dd (10.4; 4.8)	6.13 dd (10.1, 5.0)
3	3.84 m	3.84 m
4	2.05 m (2H)	2.18 m (2H)
4a	3.80 dd (13.0, 5.1)	3.75 dd (12.7, 5.0)
6	6.38 s	6.93 s
7	6.99 s	6.97 s
10	6.71 s	6.66 s
11 12	5.18 dd (7.0, 3.6)	5.20 dd (6.9, 2.7)
	3.70 dd (14.9, 7.0) 3.53 dd (14.9, 3.6)	4.12 dd (14.8, 6.9) 3.21 dd (14.8, 2.7)
OCH_2_O	5.96 s	5.96 s
	5.95 s	5.95 s
OMe	3.34 s (3H)	3.33 s (3H)
2′,6′	7.93 d (8.2) (2H)	8.00 d (8.4) (2H)
3′,5′	7.60 d (8.2) (2H)	7.62 d (8.4) (2H)
2″,6″	7.77 d (8.6) (2H)	7.77 d (8.7) (2H)
3″,5″	7.58 d (8.6) (2H)	7.59 d (8.7) (2H)

^a^ The chemical shifts are in δ values (ppm) from TMS. ^b^ 2D ^1^H, ^1^H (COSY) NMR experiment confirmed the coupling of all the protons.

**Table 2:** ^1^H NOESY NMR Data for the two 6,11-*O*,*O*′-Di-*p*-Bromobenzoyl Esters of Haemanthidine (**11** and **12**) ^a^.

11		12	
Irradiated	Observed	Irradiated	Observed
H-1	H-10, H-2	H-1	H-10, H-2
H-2	H-3	H-2	H-3
H-6	H-12A	H-6	H-4a, H-12A
H-3	H_2_-4, MeO	H-3	H_2_-4, MeO
H-11	H-12A	H-11	H-12A
H-12A	H-12B	H-12A	H-12B

^a^ The chemical shifts are in δ values (ppm) from TMS.

**Table 3:** IC_50_ values of tested compounds.

IC_50_	Hacat	A431	AGS
Lycorine	0.5 µM	0.5 µM	<0.5 µM
Haemanthamine	-	-	IC_50_ = 7.5 µM
Haemanthidine	-	-	IC_50_ = 5.0 µM

**Table 4:** *P. maritimum* alkaloids antiviral activity. EC_50_ and CC_50_ were calculated with QuestGraph IC_50_ Calculator, with concentrations ranging from 0.5 to 200 µM for HIV-1_GFP_ and from 0.05 to 200 µM for DENV_GFP_.

	EC_50_ (µM)		CC_50_ (µM)
Alkaloids	DENV_GFP_	HIV_GFP_	THP-1
Lycorine	0.39	10.90	4.61
9-*O*-Demethylhomolycorine	34.49	nd	nd
Homolycorine	65.03	nd	nd
Tazettine	12.50	120.30 *	136.90
Vittatine	15.53	nd	nd
11-Hydroxyvittatine	3.92	65.10 *	113.06
Haemanthamine	0.34	25.27	22.19
Haemanthidine	0.48	12.74	16.80
Pancracine	0.36	18.51	25.93
Obliquine	73.59	152.86 *	127.04

CC = cytotoxic concentration, EC = Effective concentration, * Incomplete inhibition; nd: not detected.

## Data Availability

Not applicable.
